# Long-Term Physical (In)Stability of Spray-Dried Amorphous Drugs: Relationship with Glass-Forming Ability and Physicochemical Properties

**DOI:** 10.3390/pharmaceutics11090425

**Published:** 2019-08-21

**Authors:** Khadijah Edueng, Christel A.S. Bergström, Johan Gråsjö, Denny Mahlin

**Affiliations:** 1 Department of Pharmacy, Uppsala University, Husargatan 3, 751 23 Uppsala, Sweden; 2 Kulliyyah of Pharmacy, International Islamic University Malaysia, Jalan Istana, 25200 Bandar Indera Mahkota, Kuantan Pahang, Malaysia

**Keywords:** long-term stability, amorphous, glass-forming ability, glass stability, physicochemical properties, crystallization, spray-drying, humidity, melt-quenching

## Abstract

This study shows the importance of the chosen method for assessing the glass-forming ability (GFA) and glass stability (GS) of a drug compound. Traditionally, GFA and GS are established using in situ melt-quenching in a differential scanning calorimeter. In this study, we included 26 structurally diverse glass-forming drugs (i) to compare the GFA class when the model drugs were produced by spray-drying with that when melt-quenching was used, (ii) to investigate the long-term physical stability of the resulting amorphous solids, and (iii) to investigate the relationship between physicochemical properties and the GFA of spray-dried solids and their long-term physical stability. The spray-dried solids were exposed to dry (<5% RH) and humid (75% RH) conditions for six months at 25 °C. The crystallization of the spray-dried solids under these conditions was monitored using a combination of solid-state characterization techniques including differential scanning calorimetry, Raman spectroscopy, and powder X-ray diffraction. The GFA/GS class assignment for 85% of the model compounds was method-dependent, with significant differences between spray-drying and melt-quenching methods. The long-term physical stability under dry condition of the compounds was predictable from GFA/GS classification and glass transition and crystallization temperatures. However, the stability upon storage at 75% RH could not be predicted from the same data. There was no strong correlation between the physicochemical properties explored and the GFA class or long-term physical stability. However, there was a slight tendency for compounds with a relatively larger molecular weight, higher glass transition temperature, higher crystallization temperature, higher melting point and higher reduced glass transition temperature to have better GFA and better physical stability. In contrast, a high heat of fusion and entropy of fusion seemed to have a negative impact on the GFA and physical stability of our dataset.

## 1. Introduction

The transformation of solids from a crystalline to amorphous form has gained attention as a formulation strategy for driving the absorption of orally administered, poorly water-soluble compounds [1,2,3]. The amorphization process involves the disruption of the long-range order of molecules in the crystal form to a solid composed of disordered molecules. The energy-optimized packing of molecules in the crystalline state governs the thermodynamic physical stability of the substance, and relatively high energy is required to disrupt the inter-molecular interactions. This energy-demanding process limits the dissolution of the crystalline material. In contrast, the absence of long-range order in an amorphous solid reduces the strength of the molecular interactions, resulting in higher kinetic solubility for amorphous solids than for their crystalline counterparts.

The success of any amorphous-based formulation is highly dependent on its ability to remain in the amorphous form. Typically, the formulation is designed to prevent, or at least delay, the conversion of amorphous solids to their more stable crystalline forms during processing, handling and storage, as well as during dissolution following oral administration. In short, crystallization of an amorphous solid is a thermodynamically driven transition that is unwanted due to its detrimental effect on the in vivo performance of amorphous formulations.

From a general formulation perspective, amorphization appears to be an applicable strategy for any drug compound with limited aqueous solubility, regardless of whether the poor solubility is solid-state or solvation-limited. In a recent review of model compounds investigated for amorphization, the studied drugs had a wide range of physicochemical properties [4]. However, the probability of obtaining a well-performing, amorphous-based formulation was dictated by the following factors: (i) the ease of which the compound forms a glassy state upon processing, (ii) the ability to remain amorphous throughout the formulation, packing, transportation, and storage processes, and (iii) the stability of the supersaturated system formed on dissolution in, for example, the gastrointestinal tract.

In earlier publications, a classification system has been proposed that categorizes organic compounds based on their glass-forming ability (GFA) and glass stability (GS) [5]. This classification system was established by subjecting a large number of drug-like organic compounds to a heat-cool-heat cycle in a differential scanning calorimeter (DSC). Based on the thermal behavior during this DSC heat-cool-heat cycle, compounds were classified as Class I if the process resulted in a crystallization upon cooling from melt, Class II if the process resulted in an amorphous form which crystallized during the second heating, or Class III if the process resulted in an amorphous form that was stable upon the second heating [5]. Since this classification system is, to some extent, heating- and cooling rate-dependent, an alternative approach has been suggested where the critical cooling rate is determined to classify the GFA of compounds [6].

This classification system has drawn a lot of attention and has resulted in the publication of a series of related studies [7,8,9,10,11]. In pharmaceutical formulation development, it has been used to indicate the suitability of amorphization as a solubility-enhancing strategy for any given compound [12]. Blaabjerg et al. [13] used a set of 18 compounds to investigate the influence of the amorphization method on GFA classification. They reported a strong correlation between GFA by melt-quenching and milling suggesting that GFA is inherent to the compound and less dependent on the amorphization method. Van Eerdenbrugh et al. [7] have investigated amorphization by solvent evaporation for a set of organic compounds and the impact of the evaporation rate on the classification of the compounds. In this case, the investigators found that the classification was, to some extent, dependent on the process chosen for amorphization; i.e., the stability of the amorphous materials produced by different techniques and/or parameter settings varied. Similar observations have been reported in studies of a single drug [14,15]. Thus, it is important, when assessing the GFA/GS class, to use the processing method likely to be used when producing the amorphous drug formulation. A better understanding of the method dependency of GFA/GS classification is needed. Further, to the best of our knowledge, there has been no reports on the relationship between glass stability classification and long-term stability studies for a large set of amorphous compounds under both dry and humid conditions.

Spray-drying is one of the most commonly used methods for producing amorphous formulations, both in academic research and pharmaceutical manufacturing [4,16,17]. In this study, we made a GFA/GS classification by processing 26 drug compounds with spray-drying and compared the results to those obtained from classification by melt-quenching. We then undertook long-term stability tests (six months) at humid and dry conditions and investigated the correlation of the resulting physical stability to the GFA/GS classifications. Finally, we explored potential relationships between the physicochemical properties (calculated and measured), the GFA/GS class, and the long-term physical stability.

## 2. Materials and Methods

### 2.1. Materials

A structurally diverse dataset (*n* = 26), composed of compounds previously classified as glass formers (GFs) when prepared by melt-quenching, solvent evaporation, or spray-drying methods, was included in the study [5,7,8,9,10,18]. Only compounds supplied in their free crystalline form (i.e., no salts, solvates, hydrates, etc.), as confirmed by DSC analysis, were used.

Metolazone was purchased from API Chemical (Hangzhou, China). Acetaminophen, bezafibrate, clofoctol, chlorpropamide, dimethyl sulfoxide, glibenclamide, glipizide, hydrocortisone, sodium hydroxide, sodium phosphate, sodium chloride, sulfamerazine, sulfathiazole, and tinidazole were supplied by Sigma-Aldrich (Steinheim, Germany). Aripiprazole, cinnarizine, clotrimazole, droperidol, d-salicin, fenofibrate, flurbiprofen, hydrochlorothiazide, ibuprofen, ketoconazole, ketoprofen, prednisone, and probucol were obtained from Toronto Research Chemical (Toronto, ON, Canada). Indapamide and procaine were purchased from Tokyo Chemical Co. Ltd. (Tokyo, Japan). Acetone was supplied by Merck (Darmstadt, Germany), ethanol by Solveco (Rosersberg, Sweden), and phosphorus pentoxide by VWR (Leuven, Belgium). All of the chemicals used in this study were of either pharmaceutical or analytical grade with a specified purity of ≥ 97%.

### 2.2. Preparation of Samples

#### 2.2.1. Melt-Quenching Using Differential Scanning Calorimetry

The amorphous forms of the drug compounds were prepared via melt-quenching for a GFA/GS assignment. The melt-quenched samples were not included in the subsequent stability studies. The in situ melt-quenching method followed a procedure previously employed by Alhalaweh et al. [9]: 1–5 mg of the compound was weighed into an aluminum pan with a non-hermetic lid. The sample was heated to 2 °C above the measured melting point (T_m_) at a constant rate of 10 °C/min in a Q2000 differential scanning calorimeter (TA Instruments Co., New Castle, DE, USA). To ensure complete melting, the heated compound was held isothermally for 3 min at this elevated temperature after which it was cooled to −70 °C at a rate of 20 °C/min. Immediately thereafter, the cooled sample was heated to 10–20 °C above the T_m_ at a rate of 20 °C/min. The melt-quenching was performed in at least two replicates. The GFA classifications by melt-quenching was done based on the following behavior in the DSC: crystallization was observed upon cooling (Class I), crystallization observed during second heating (Class II), and no crystallization observed either upon cooling or during second heating (Class III).

#### 2.2.2. Spray-Drying

A standard spray-drying method was used to prepare the samples. In order to investigate the ability of the compounds to become amorphous from spray-drying we (i) standardized the process settings without individual customization for any compound and (ii) spray-dried the compounds in their neat form, without the addition of any excipients to assist amorphization and/or stabilization.

The spray-drying solutions were prepared by dissolving the crystalline powders as supplied in ethanol, acetone, or a mixture of both at 90:10% *w*/*w*. The selection of solvent was based on the solubility of the compound in the solvent system. After complete dissolution of the powder, the solution was filtered using a disposable bottle-top filter with 0.45µm pores to remove any debris or particulate impurities.

The solutions were then spray dried in a Büchi B-290-Mini Spray Dryer with an inert loop (Büchi Laboratoriums, Flawil, Switzerland) using a previously optimized generic method suitable for spray-drying of many different compounds. The following process settings were used [18]: inlet temperature 55 °C, outlet temperature between 38 °C and 40 °C, spray solution flow rate 4 mL/min, air flow rate 50 mL/min, and aspiration rate 75% of the maximum flow. The spray-dried material was divided into two aluminum containers and dried overnight in a desiccator connected to a continuous vacuum pump at room temperature (22 °C) prior to various solid-state analyses and the physical stability study (details presented in Section 2.3 below). The GFA/GS classification of spray-dried material is described in Section 2.4.2 below.

### 2.3. Physical Stability upon Storage

After overnight drying, a fraction of each spray-dried material was weighed into open DSC pans (TA standard aluminum pan). The aluminum containers and DSC pans containing the spray-dried samples were stored under both dry (<5% RH) and humid (75% RH) conditions at 25 °C for six months (168 days) or until they completely transformed into their crystalline form, whichever came first. Sampling of the bulk powder in the open aluminum container was done for Raman spectroscopy and polarized light microscopy (PLM) measurements and pre-weighted samples in DSC-pans for DSC analysis. To make the experimental workload feasible, and based on the fact that solid-state changes are more likely to occur under humid storage conditions [19,20], the samples stored at 75% RH were primarily monitored. Therefore, samples were withdrawn from the compounds stored at 75% RH at 1, 2, 7, 14, 28, 84, and 168 days to assess their solid-state characteristics. If solid-state changes were observed in the samples at 75% RH, solid-state characterization was also carried out on samples stored at <5% RH. Samples that were fully crystalline at the first assessment directly after spray drying were not subjected to stability tests and further characterizations were not carried out.

### 2.4. Solid-State Characterization

#### 2.4.1. Powder X-ray Diffraction (PXRD)

The X-ray diffractograms of the crystalline compounds as received from the manufacturer, and the spray-dried samples after drying were collected using a Twin-Twin Diffractometer (Bruker, Coventry, UK) measuring in Bragg-Brentano geometry and equipped with a sample rotator. The diffraction pattern was collected between 2*θ* of 5° and 40° with a scanning step of 0.01° and a sample rotation of 40 rpm. During the measurement, the samples were exposed to Cu Kα radiation (λ = 1.542 Å), generated by a rotating copper anode working at 40 kV and 40 mA. Primary and secondary divergence slits of 0.40 and 2.48 mm were used. The solid material was analyzed without further processing, except for some crystalline compounds that were too coarse to be analyzed. These compounds were gently ground with a mortar and pestle to produce a smooth surface on the Si-plate sample holder and to minimize the orientation effects caused by large crystals.

#### 2.4.2. Modulated Differential Scanning Calorimetry (MDSC)

The thermograms obtained from the modulated differential scanning calorimeter in this study served three main purposes. They were used (i) to obtain the T_m_ and heat of fusion (ΔH_f_) of the crystalline materials, (ii) to monitor the solid-state changes of the spray-dried stability samples over time by monitoring changes in heat capacity (ΔC_p_), T_g_, T_c_, and T_m_, and (iii) to determine the GFA classes of the spray-dried samples. The samples withdrawn at each time point, were analyzed at least in two replicates.

##### Determination of Melting Point and Heat of Fusion

To determine the T_m_ and ΔH_f_, 1–5 mg of each sample was weighed into a standard aluminum pan with a non-hermetic lid. In the MDSC analysis, the samples were first equilibrated at either −70 °C or 0 °C; the temperature was chosen to allow a baseline of at least 20 °C below the expected T_g_ of the samples. For compounds with a T_g_ ≥ −50 °C, −70 °C was used, whereas compounds with T_g_ ≥ 20 °C were equilibrated at 0 °C. The samples were then heated at a rate of 3 °C/min to 20–30 °C above the T_m_, with temperature modulation of ±1 °C every 60 s. The T_m_ and ΔH_f_ were obtained from the total heat flow signal of the MDSC thermograms.

##### Solid-State Characteristics of Spray-Dried Stability Samples

Using the same MDSC protocol described in previous section, the ΔC_p_, T_g_, T_c_, and T_m_ were monitored for the samples withdrawn at different time points through the stability. During storage at 75% RH, it was highly likely that water was absorbed by the spray-dried samples. Therefore, prior to the MDSC runs, drying of the stability samples were required to remove the water to obtain an accurate value of ΔC_p_. The samples were dried in the DSC instrument by heating them in a standard aluminum pan without a lid to 20 °C below the T_g_ and then holding them isothermally at that temperature for 30 min. The weight of the sample after drying was recorded and used for the MDSC runs where the values for ΔC_p_, T_g_, T_c_, and T_m_ were obtained.

##### Determination of GFA/GS Classes of Spray-Dried Samples

The GFA/GS classes of the spray-dried samples were assigned based on their thermal behavior upon heating in the DSC (no information on crystallization upon cooling of melt as in situ melt-quenching in DSC). Classification was done as Class I—if only a melting was detected, Class II—if crystallization occurred after above the T_g_ followed by a melting, and Class III—if a glass transition was detected without subsequent crystallization and melting event.

#### 2.4.3. Raman Spectroscopy

An Rxn-2 Hybrid Raman Spectrometer (Kaiser Optical System Inc., Ann Arbor, MI, USA) equipped with a laser (λ = 785 nm, power = 400 mW) and a fiber-optic PhAT probe was used to characterize and monitor the solid-state forms and changes of the supplied crystalline materials, the spray-dried materials (after overnight drying), and the samples withdrawn at different time points in the stability study. Approximately 5–20 mg of each sample was placed on an aluminum sample holder. The spectra were recorded in the wavenumber range of 100–1890 cm^−1^ and processed to allow semi-quantitative analysis of the crystallization rate of the stored materials. The processing protocol of the spectra and the semi-quantification of crystallinity are described in detail below.

Selection of spectral region and spectra pre-treatment protocol: A wavenumber region with relatively large differences between the crystalline and spray-dried samples with a constant baseline was selected for analysis. Thereafter, endpoints where both crystalline and spray-dried spectra showed a valley and where the intermediate background-corrected spectral values were not negative were chosen. The latter requirement was in some cases not met, but in these cases, the corrected spectral values were not overly negative, and the sub-regions in question were small. The spectral background was estimated as a straight baseline between the endpoints of the spectral region under analysis, and the background-corrected spectrum was calculated by subtracting the baseline values at the respective wavenumber. After the baseline correction, each spectrum was normalized for the measured intensities by dividing the spectrum by the sum of intensities of the background-corrected spectrum (which is equivalent to the area under the curve, numerically determined by the trapezoidal method).

Analysis by classical least squares: The measured spectra, after background correction and normalization, were expected to be equivalent to the weighted sum of the proportional crystalline and spray-dried spectra, giving the following relationship (Equation (1)):

𝑦̅_𝑠𝑦𝑛𝑡ℎ_ = 𝑓_*CR*_∙𝑦̅_𝐶𝑅_ + 𝑓_𝑆𝐷_∙𝑦̅_𝑆𝐷_ = (1 − 𝑓_𝑆𝐷_)∙𝑦̅_𝐶𝑅_ + 𝑓_𝑆𝐷_∙𝑦̅_𝑆𝐷_ (1)

where 𝑓_*CR*_ and 𝑓_𝑆𝐷_ are the weighted factors of the spectra of spray-dried and crystalline samples and 𝑦̅_𝐶𝑅_ and 𝑦̅_𝑆𝐷_ are the vector representations of the normalized crystalline sample (CR) spectrum and the normalized spray-dried sample (SD) spectrum, and 𝑦̅_synth_ the vector representation of the resulting synthesized spectrum. The factor 𝑓_𝑆𝐷_ was determined by least square curve fit of Equation (1) to the measured spectra and 𝑓_CR_ was determined as 1 − 𝑓_𝑆𝐷_.

The quantities 𝑓*_CR_* and 𝑓*_SD_* should not be strictly interpreted as the fraction of crystalline and untransformed spray-dried species in the sample but can be used as rough estimates of these fractions. For quantification purposes, additional studies and modification of Equation (1) are required [21]. In our study, the obtained 𝑓_𝑆𝐷_ values were regarded as a rough approximation of the real fraction and were used to rank the samples with respect to their amorphous content; this was considered sufficient for the current aim. The data pre-treatment, as described above, and the curve fitting to determine 𝑓_𝑆𝐷_ were undertaken using a Microsoft Excel spreadsheet developed in-house, where the Microsoft Excel Solver (Microsoft Office 2016, Redmon, WA, USA) function was used for the least-square curve-fit.

#### 2.4.4. Polarized Light Microscopy

Micrographs of the crystalline compounds as supplied, the freshly spray-dried materials, and the stability samples withdrawn at the time points specified in Section 2.3 were collected using an Olympus BX51 polarizing microscope (Olympus Corporation, Tokyo, Japan). The samples were dispersed in oil on a microscope slide immediately before the analysis to improve image quality and clarity.

### 2.5. Combining the PXRD, DSC, Raman Spectroscopy, and PLM Analyses

The solid-state forms (i.e., amorphous, crystalline, and mixture of amorphous and crystalline) of the unprocessed crystalline (as received from the manufacturer) and freshly spray-dried samples were characterized using a combination of PXRD (Bruker, Coventry, United Kingdom), Raman spectroscopy (Kaiser Optical System Inc., Ann Arbor, MI, USA), DSC (TA Instruments Co., New Castle, DE, USA), and PLM (Olympus Corporation, Tokyo, Japan). These initial characterizations served as the references to monitor the solid-state changes of the stored samples over time. At every sampling point, Raman spectroscopy was used for the semi-quantification of amorphous and/or crystalline content of the samples, and the results were confirmed with DSC and PLM data.

### 2.6. Univariate and Multivariate Analysis

The properties used for the statistical analysis are listed in Table 1. Univariate analysis was used to reveal any strong relationships between the GFA, the long-term physical stability of the spray-dried compounds, and the physicochemical properties (including thermal properties).

In addition to the univariate analyses, multivariate analysis was used to capture the potential influence of the physicochemical properties (individually or in combination) on the GFA and long-term physical stability. For this purpose, principal component analysis (PCA) was carried out with SIMCA 15 (Umetrics, Umeå, Sweden). The physicochemical properties were mean-centered and scaled to unit variance prior to the PCA. Properties that were skewed >1.5 after this were excluded from further analysis. PCA was used to explore clustering and trends of compounds and descriptors whereas partial least squares—discriminant analysis was used to explore how the different physicochemical properties were related to GFA and GS. For the GFA, compounds that were either glass formers (GFs) and non-glass formers (nGFs) were compared. The analysis was thereafter extended to further discriminate the compounds based on whether they were fully amorphous (A), a mixture of amorphous and crystalline (AC), or fully crystalline (C) upon spray-drying. For analysis of physical stability on storage, the compounds were classified as stable GFs and unstable GFs.

## 3. Results and Discussion

### 3.1. Selection of the Dataset

The 26 compounds selected for inclusion in the study were diverse in their physicochemical properties to obtain as general conclusions as possible. The main focus was on poorly water-soluble drugs, but we also included compounds that have satisfactory solubility from an administered dose perspective, although amorphization is a formulation strategy mainly targeting poorly water-soluble compounds. Various physicochemical properties of importance for GFA and GS was also considered in the selection process [22].

Table 1 summarizes the GFA and GS classes determined from in situ melt-quenching in a DSC with standard heating and cooling rates [9], and the main physicochemical properties of the selected compounds that have been previously linked to GFA and GS. Ten of the 26 compounds were classified as unstable GFs (Class II), while the remaining 16 compounds were classified as stable GFs (Class III) by the melt-quenching method.

### 3.2. Glass-Forming Ability and Glass Stability

In this study, a compound was classified as a GF when amorphous content in the spray-dried sample was detected using any or all of the solid-state characterization methods. This means that the resulting material could have been either completely amorphous or partly amorphous (i.e., a mixture of amorphous and crystalline substances). A compound was considered to be an nGF if it was fully crystalline after spray-drying.

With regard to the storage stability, a sample was considered to be a stable GF if there was no measurable reduction in the amorphous content after 168 days of storage under the two conditions described in Section 2.3 compared to the initial sample. This was only applicable to compounds that were fully amorphous after spray-drying. In contrast, a compound was considered to be an unstable GF if (i) it was partially amorphous after spray-drying, (ii) the amorphous content decreased over the 168 days of storage, and/or (iii) the amorphous content converted completely to crystals, as indicated by the solid-state analyses. It should be noted that some compounds crystallized into another polymorph than initially obtained from the supplier, when converting from the amorphous state during storage.

**Figure 1:**
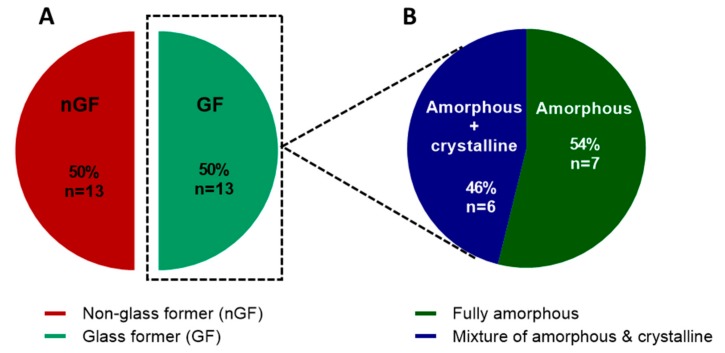
Pie charts showing (**a**) the glass-forming ability (GFA) of the model compounds produced via a standard spray-drying method and (**b**) the solid-state forms of the glass formers (GFs). The spray-dried compounds were classified as either non-glass formers (nGFs) or GFs. The GFs were further divided as either producing fully amorphous or a mixture of amorphous and crystalline material.

**Figure 2:**
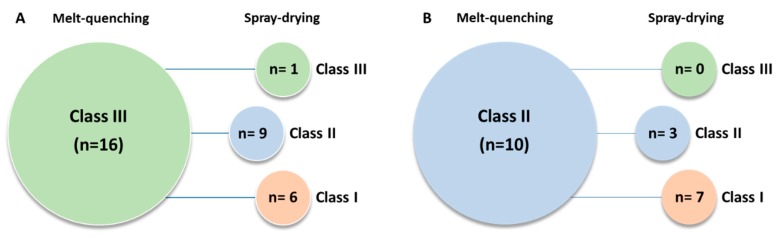
The number of compounds in glass-forming ability/glass stability (**A**) Class III and (**B**) Class II according to in situ DSC melt-quenching compared to glass-forming ability/glass stability classes according to spray-drying, respectively. Pink, blue, and green represent Classes I, II, and III, respectively.

All of the selected compounds have been classified as GFs in previous studies [5,7,8,9,10], according to in situ melt-quenching DSC data. Fifty percent (*n* = 13) of the dataset were classified as GFs when spray-drying was used instead of melt-quenching, while another 50% (*n* = 13) were classified as nGFs (Figure 1a). Of the 50% that were classified as GFs, only seven were completely amorphous after spray-drying, while six were a mixture of amorphous and crystalline material (Figure 1b). Indapamide, metolazone, glibenclamide, hydrochlorothiazide, hydrocortisone, ketoconazole, and sulfathiazole were completely amorphous, while prednisone, aripiprazole, glipizide, droperidol, clotrimazole, and probucol were a mixture of amorphous and crystalline material after spray drying. Hence, the GFA results after spray-drying shown in Figure 2 are significantly different from those after the in situ melt-quenching method was used to produce the amorphous form of the compounds.

Figure 2 summarizes the GFA/GS classes of the compounds and shows how the classification was dependent on if spray-drying or melt-quenching was used. The melt-quenching method resulted in 10 Class II and 16 Class III amorphous forms. However, of the 16 Class III melt-quenched amorphous materials, only one remained in the same class when the spray-dried method was used; nine of them were categorized as Class II by spray-drying (Figure 2a). Six of the compounds assigned as Class III with the melt-quenching method were completely crystalline after spray-drying, hence being classified as Class I. Of the 10 compounds classified as Class II by melt-quenching, only three retained the same class upon spray-drying, whereas the other seven became Class I by spray-drying (Figure 2b). None of the compounds being Class II by melt-quenching were classified as Class III by spray-drying.

The GFA/GS classification by melt-quenching largely agree with previous reports where classification by in situ DSC melt-quenching had been done [5,8,9]. It is apparent from our data that spray-drying is much less likely than melt-quenching to generate a material in an amorphous form. Interestingly, none of the spray-dried compounds were up-classified compared with the melt-quenching classification. In fact, all except four of the model compounds were down-classified. This suggests that the GFA/GS classification is strongly influenced by the processing method employed.

#### 3.2.1. Influence of Preparation Method vs. Compound GFA/GS

The impact of different methods of preparation on the GFA of specific compounds is known. Theoretically, GFA is dependent on the cooling rate from melt or evaporation rate of a solvent. In one study, the effects on the GFA/GS classes of compounds prepared by in situ DSC melt-quenching were compared with solvent evaporation methods [7]. It was also found that retention, promotion, or demotion of the GFA/GS class depended on method and the solvent used. This is somewhat in contrast to what we have found earlier—that for a set of compounds, the GFA for most compounds was independent of preparation method used (melt-quenching, spray-drying, and mechanical activation) [11]. All these suggest that, the likelihood of rendering a drug compound amorphous is primarily dependent on the “inherent” GFA and GS properties, but many compounds are also sensitive to conditions at which glass formation happens. For compounds with very high GFA/GS (e.g., indapamide), the method of preparation is of minor importance, as long as standard conditions for amorphization are used.

Compared to evaporation at ambient temperature and rotary evaporation, the spray-drying method used in our study provided relatively rapid evaporation, which give rise to hollow and porous particles. The standardized settings of the spray-drying equipment and powder collection by the cyclone in the instrument assure similar particle size range of the different compounds. Any particles surface area variations influencing re-crystallization tendency should hence be minimized. It cannot however be excluded that the introduction of a relatively large particle surface area is contributing to a general reduction of GFA/GS of the compounds in spray-dried form compared to when melt-quenched.

**Figure 3:**
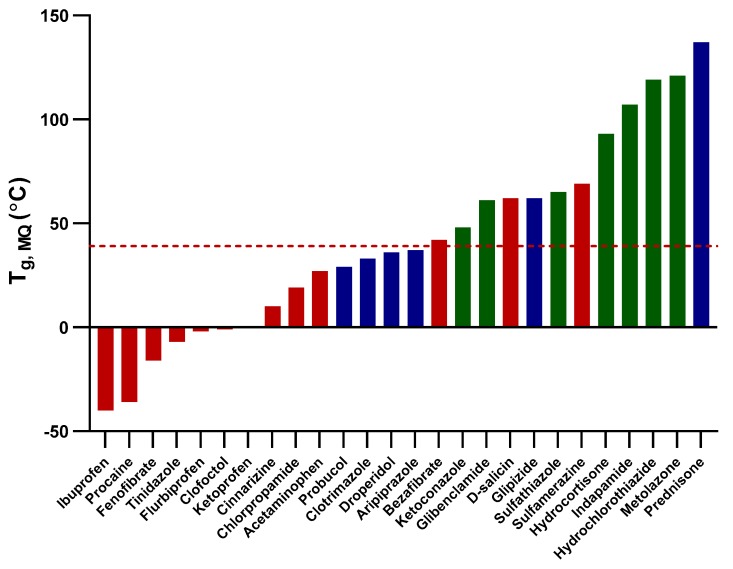
A bar chart indicating the glass transition temperatures (T_g, MQ_) of the melt-quenched studied compounds in relation to the average spray-drying outlet temperature (red dashed-line; 39 °C). The solid-state forms of the compounds on spray-drying are represented by different colors: red represents fully crystalline, blue represents a mixture of amorphous and crystalline, and green represents fully amorphous compounds.

Figure 3 shows the T_g_ of the melt-quenched model compounds in relation to the outlet temperature in the spray-drying process. The figure displays a general trend of the spray-dried products based on whether the T_g_ was below, close to or above the outlet temperature. Compounds having T_g_ below the outlet temperature were prone to be spray-dried as fully crystalline, whereas those with T_g_ that were close or similar to the outlet temperature tended to be spray-dried as mixtures of amorphous and crystalline. Compounds with T_g_ higher than the outlet temperature more often produced fully amorphous spray-dried materials. This shows another important difference between amorphization by melt-quenching and spray-drying. In melt-quenching, the compound is formed upon cooling to a temperature well below T_g_, whereas the drying temperature is limiting the success for amorphization of compounds with low T_g_ via spray-drying_._

It was expected that compounds with T_g_ below the outlet temperature would be spray-dried in the crystalline form. It was, however, more surprising that compounds with T_g_ higher than the outlet temperature (d-salicin, bezafibrate, and sulfamerazine) were completely crystalline upon spray-drying. It was also interesting to see that glipizide and prednisone, with T_g_ 23 °C and 98 °C above the outlet temperature, did not become fully amorphous. For these compounds, selection of the solvent, and resulting hydrogen-bond interaction, might have a relatively more important contribution to the solid-state form of the spray-dried output.

**Figure 4:**
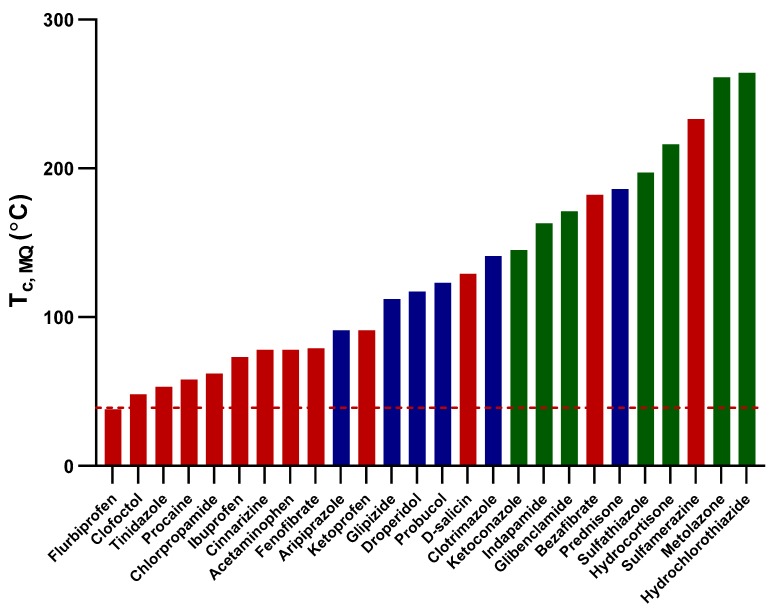
A bar chart demonstrating the crystallization temperatures of melt-quenched (T_c, MQ_) of the study compounds in relation to the average spray-drying outlet temperature (red dashed-line; 39 °C). The solid-state forms of the compounds on spray-drying are represented by different colors: red represents fully crystalline, blue represents a mixture of amorphous and crystalline, and green represents fully amorphous compounds.

A similar trend was observed in Figure 4, which demonstrates the melt-quenched T_c_ of the compounds compared to the average outlet temperature of the spray-drying process. Compounds with lower T_c_ values were spray-dried as crystalline, whereas those with relatively higher T_c_ values were spray-dried either as an amorphous-crystalline mixture or as fully amorphous substances.

An amorphous solid produced via different methods or method parameters can have variable degrees of amorphicity (“degrees of disorder”) [15,23]. Theoretically, a specific amorphous compound in different disordered states, with different glass densities, will possess different physical stability. It is possible to distinguish the degree of disorder by comparing the thermal parameters such as T_g_ and T_c_, the shape of the PXRD halo patterns, atomic pair-wise distribution function of a Fourier transformation of PXRD diffractograms, terahertz spectroscopy, and solid-state nuclear magnetic resonance [14,15,24,25,26]. These differences in crystallization tendency may be attributed to different degrees of amorphicity and/or the presence of seed crystals [27]. In our study, melt-quenched amorphous clotrimazole, hydrochlorothiazide, and prednisone had profoundly different T_g_ values from the spray-dried forms, which may indicate different degrees of disorder induced by the different preparation techniques.

### 3.3. Long-Term Physical Stability

The physical stability of compounds that formed either fully amorphous substances or a mixture of amorphous and crystalline substances upon spray-drying were studied long term as described in Section 2.3 No further evaluations were made of the 13 compounds that were completely crystalline after spray-drying (Table S1).

**Figure 5:**
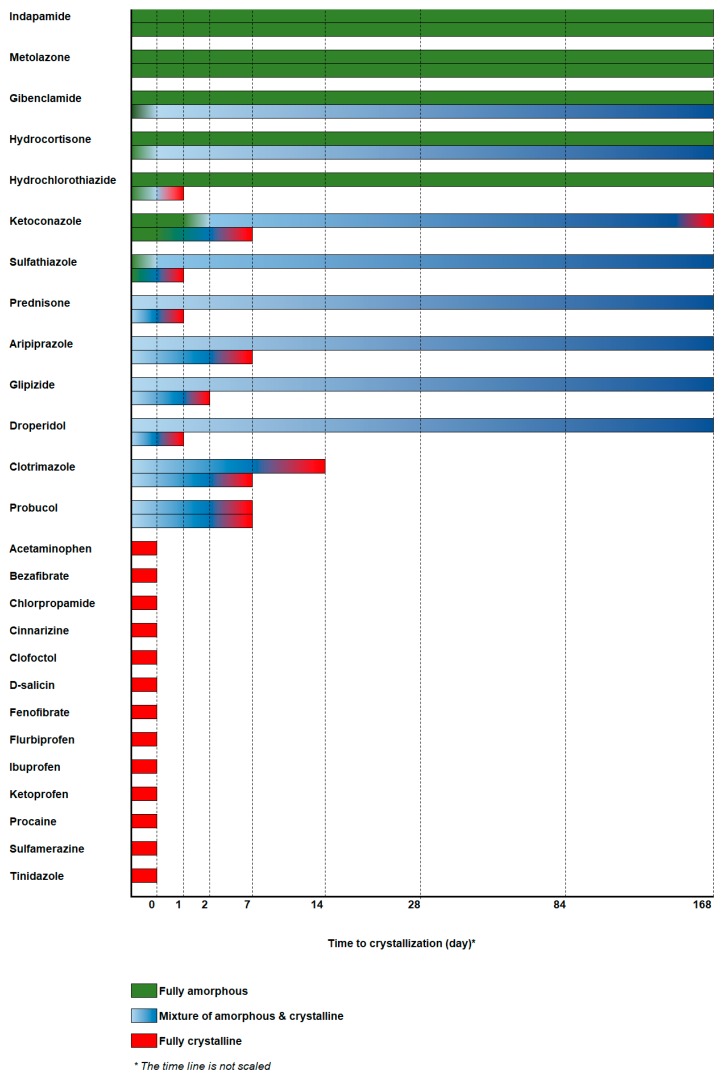
The six-month physical stability of the spray-dried compounds. The stability of each compound is represented by color gradients over the study period: fully amorphous (green), different ratios of amorphous and crystalline mixtures (light to dark blue), and fully crystalline (red). The top and bottom bars for each compound represent the stability under dry conditions (<5% RH) and humid conditions (75% RH), respectively.

The overall long-term physical stability profile of the compounds is illustrated in Figure 5 and Table S1. The compounds can be categorized as being stable under both dry and humid conditions, stable under dry conditions but unstable under humid conditions, or unstable under both storage conditions. It is also interesting to highlight compounds that nucleate rapidly but grow very slowly when stored under dry conditions, even in the presence of seed crystals.

Indapamide and metolazone remained stable in the amorphous form regardless of the storage conditions, throughout the course of the study. These two compounds were not affected by humidity. This is not surprising, as both have high spray-dried T_g_ and T_c_. Glibenclamide, hydrocortisone, and hydrochlorothiazide were stable under dry conditions but crystallized at different rates in the presence of humidity. While glibenclamide and hydrocortisone crystallized only minimally (f_CR_ = 0.06 and 0.11, respectively) under humid conditions, hydrochlorothiazide became completely crystalline in less than a day, suggesting rapid crystallization when exposed to moisture. This may reflect the different tendencies of these compounds to absorb and interact with water molecules and cause de-stabilization, given that all of them had reasonably high spray-dried T_g_ and T_c_. It is also noteworthy that, of these three compounds, hydrochlorothiazide is the most hydrophilic (logP = −0.1), which is probably why it formed crystals the most rapidly of the three at 75% RH; hydrocortisone and glibenclamide (logP of 1.6 and 4.8, respectively) are relatively less hydrophilic and hence, would not interact with water to the same extent. The presence of water has been reported to increase the intermolecular pore size, i.e., the free volume of the amorphous structure, leading to an increased molecular mobility within the system (the plasticizing effect) [19,28]. Water primarily affects the relaxation associated with cooperative molecular motion. To the best of our knowledge, no extensive studies have compared the water activity and sensitivity of a large number of pharmaceutical compounds, probably because of the complexity of and long timelines needed for such studies. We also lack data on humidity sorption to make any conclusions but is of interest for further studies.

Sulfathiazole showed an interesting crystallization behavior. At 75% RH, complete crystallization of sulfathiazole was rapidly achieved (in less than a day). Crystallization was also initiated rapidly under ambient conditions. For instance, the crystalline peak intensity almost doubled in the second of two PXRD runs taken 20 min apart (Figure S1). This was confirmed by the appearance of birefringence on PLM micrographs of the sulfathiazole samples stored under ambient conditions compared to the freshly spray-dried samples (Figure S2). This indicates that crystallization of amorphous sulfathiazole was also triggered by modest exposure to humidity in the surrounding environment. However, under dry conditions, although sulfathiazole started to crystallize rapidly, it was not entirely crystallized at the last sampling point. Less than half of the initial amorphous sulfathiazole was crystallized (f_CR_ = 0.38). This shows that under dry conditions, sulfathiazole nucleates rapidly but the crystals grow slowly. The hydrophilicity of sulfathiazole (logP = 0.1) also indicated that it would crystallize rapidly in the presence of humidity. A strong affinity for and interaction with water can accelerate both nucleation and the crystal growth process.

In contrast, probucol, which was spray-dried as an amorphous-crystalline mixture, crystallized at a comparable rate under both dry and humid conditions, which indicates that the crystallization process was not influenced by the presence of and/or interaction with water molecules during exposure. Because probucol is highly lipophilic (calculated logP = 11.3), it was very unlikely that crystallization would be induced and controlled by its interaction with water molecules. However, it could have been influenced by its physicochemical properties such as T_g_ (26 °C), which was very close to the storage temperature (25 °C), where molecular mobility therefore would be enhanced.

Aripiprazole and droperidol underwent rapid crystallization after around seven days and one day, respectively, of storage at 75% RH. However, they crystallized very slowly under dry conditions, despite having been spray-dried as amorphous-crystalline mixtures. The presence of seed crystals in the sample did not seem to induce rapid crystal growth. Aripiprazole and droperidol have T_g_ of 31 °C and 33 °C, respectively; these were not strikingly different from the T_g_ of probucol, but the drugs exhibited superior stability under dry conditions. The crystallization of these two compounds was accelerated by an interaction with water molecules upon exposure to humidity, which would have increased their molecular mobility.

### 3.4. Relationship between GFA/GS Classifications and Storage Stability

The GFA/GS classification from melt-quenching and spray-drying is found in Table 1. From the discussion in Section 3.2.1, it can be understood that, the discrepancy between the classification derived from melt-quenching and spray-drying is due to compounds with T_g_ and T_c_ close to the outlet temperature become crystalline no matter if it is a Class III by melt-quenching. For the few compounds with higher T_g_ and T_c_, still becoming Class I on spray-drying, the explanation probably lies in a solvent-interaction effect. This shows the importance of using the right amorphization method when judging a compound suitability for amorphous formulation.

There seem to be a strong correlation between the stability at dry condition and the GFA/GS classification by spray-drying. The Class I compounds are naturally not relevant here, since they did not transform into amorphous upon spray-drying. The only Class III compound (i.e., indapamide) was stable upon storage over the six months (Figure 5). The Class II compounds may first appear unpredictable. However, it can be seen that all Class II compounds with T_c_ above about 120 °C became fully amorphous upon spray-drying (Figure 4) and showed good stability upon storage (Figure 5). All Class II compounds with lower T_c_ are mostly partially crystalline after spray-drying, and they showed different rate of crystallization upon storage for six months (Figure 5). A similar principle seems to be applicable to predict storage-stable-compounds by using data from GFA/GS classification by melt-quenching. All storage-stable-compounds were classified as Class III by melt-quenching. The difference between them and all other Class III compounds is that they all display a T_g_ above 90 °C. The only compound being falsely predicted by using this principle is prednisone, with a T_g_ of 137 °C, still crystallizing upon storage for six months. The storage at humid conditions was not possible to predict with any certainty. This is probably due to differences in interaction with and sorption of water. Further studies of this matter are of importance to understand sensitivity to humidity better.

### 3.5. Role of the Physicochemical Properties on the GFA and Long-Term Physical Stability

#### 3.5.1. GFA vs. Physicochemical Properties

Based on the findings of this study, we further investigated (i) whether the ability of the selected compounds to become amorphous upon spray-drying was related to any of their physicochemical properties and (ii) whether the long-term stability of the compounds was related to any of their physicochemical properties. For this purpose, we evaluated several physicochemical properties that have been reported as important determinants of GFA and GS as well as properties that may have an effect on the interaction with water (shown in Table 1) by performing both univariate and multivariate analysis.

None of the physicochemical properties were strongly correlated with the GFA (Figure S3). Nevertheless, a weak positive correlation between GFA and MW, T_m_, T_g_, and T_rg_ was observed. The ΔH_f_ and ΔS_f_, in contrast, had a negative impact on the ability of compounds to form glass, and the remaining physicochemical properties did not seem to explain the GFA of the compounds. The same correlation pattern was also found when similar analysis were performed for compounds classified according to GFA classes proposed by Baird et al. [5] (Figure S4). The GFA Class II and III compounds tended to have slightly higher MW, T_m_, T_g_, and T_rg_ but lower ΔH_f_ and ΔS_f_ than the Class I. In our dataset, the physicochemical properties of GFA Class II and III could not be discriminated due to that only one compound was spray-dried as Class III. The weak correlation suggests that the GFA of the compounds are not strongly linked to any of the single individual physicochemical property included in this study. To investigate whether stronger relationships were obtained when several physicochemical properties were included, a multivariate discriminant analysis was performed. It should be noted that although we had access to one of the largest publically available datasets, it is still relatively small for this kind of analysis and was only performed to explore trends. It was found that the GFA classification separating GFs from nGFs resulted in a one principal component (PC) model with *R*^2^ of 0.66, *Q*^2^ of 0.61 and only two physicochemical properties were found to be important. These were the T_g_ and Mw, both being positively related to GFs in agreement with what was observed based on the univariate data analysis. Hence, it can be concluded that based on all properties that were explored these have the strongest influence on the GFA of the studied dataset.

**Figure 6:**
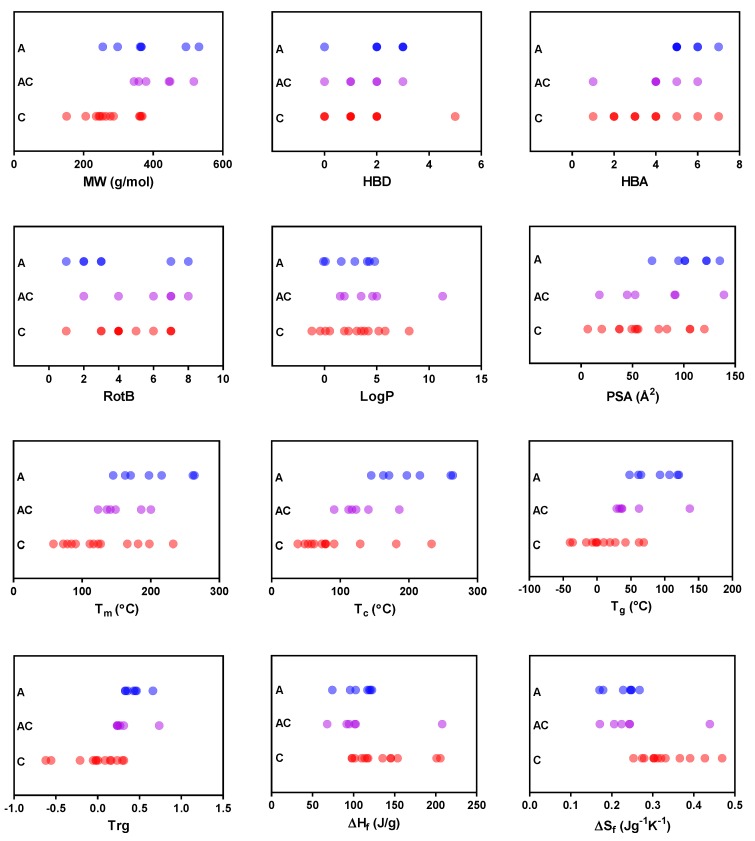
Univariate analysis of the relationships between the glass-forming ability of the compounds upon spray-drying and selected physicochemical properties. The physicochemical properties presented are molecular weight (MW), number of hydrogen bond donors (HBD), number of hydrogen bond acceptors (HBA), number of rotatable bonds (RotB), logP, polar surface area (PSA), melting point (T_m_), crystallization temperature (T_c_), glass transition temperature (T_g_), reduced glass transition temperature (T_rg_), heat of fusion (ΔH_f_), and entropy of fusion (ΔS_f_). The compounds are categorized as fully amorphous (A), a mixture of amorphous and crystalline (AC) or fully crystalline (C).

In order to further evaluate the GFA robustness of the compounds (i.e., their ability to transform to a fully amorphous form), the same univariate analysis was carried out. For this analysis, however, the GFs were divided into two sub-groups: (i) those that became fully amorphous and (ii) those that produced a mixture of amorphous and crystalline forms upon spray drying. Figure 6 shows that the relationship with some physicochemical properties was better discriminated when the GFs were separated in this way. For instance, there was a trend for the fully amorphous group to have a relatively higher PSA, T_m_, and T_c_, which was not clearly distinguishable when GF and nGF were used as the dependent variables. The outliers from the GF group were also separated for ΔH_f_ and ΔS_f_ when the GFs were divided into those that formed a mixture of amorphous and crystalline substances and those that formed fully amorphous substances, giving a clearer correlation between these two properties. Similar to the outcomes of univariate analysis in Figure S3, the GFA seemed to be better for compounds with larger MW and higher T_g_ and T_rg_, and worse for compounds with higher ΔH_f_ and ΔS_f_. When the multivariate discriminant analysis was performed, a one PC model was obtained (*R*^2^ of 0.63, *Q*^2^ of 0.60) this time based on three descriptors. These were T_c_ and T_g_, both being positively related to fully amorphous, and ΔS_f_ negatively influencing the compound to become fully amorphous. Hence, it can be concluded that based on all properties that were explored these were the ones have the strongest influence on the ability of compounds to be spray-dried in fully amorphous form.

#### 3.5.2. Long Term Physical Stability vs. Physicochemical Properties

**Figure 7:**
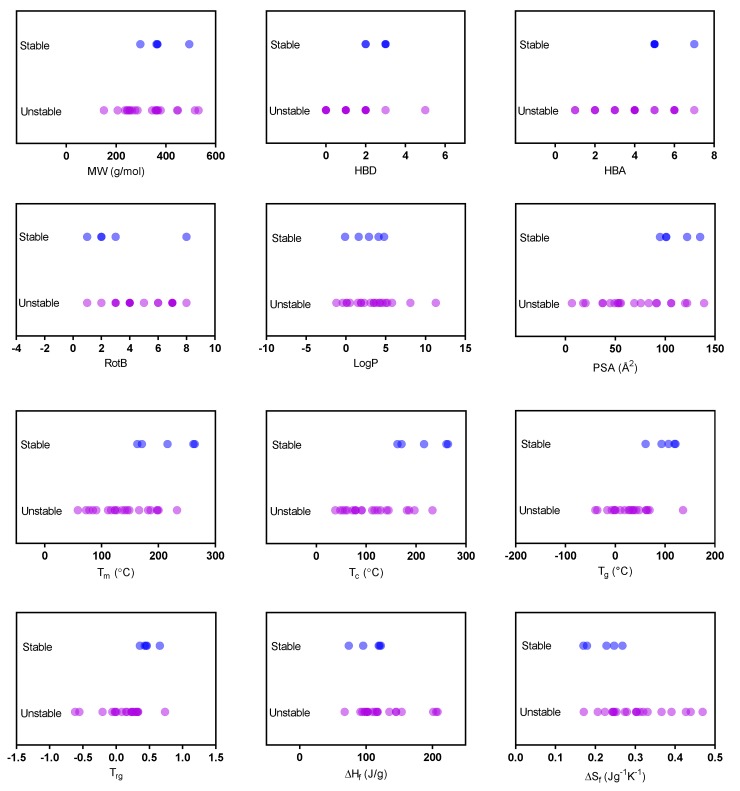
Univariate analysis of the relationships between the long-term physical stability of the spray-dried compounds stored for 168 days (6 months) under dry conditions (<5% RH) and selected physicochemical properties. The physicochemical properties presented are molecular weight (MW), number of hydrogen bond donors (HBD), number of hydrogen bond acceptors (HBA), number of rotatable bonds (RotB), logP, polar surface area (PSA), melting point (T_m_), crystallization temperature (T_c_), glass transition temperature (T_g_), reduced glass transition temperature (T_rg_), heat of fusion (ΔH_f_) and entropy of fusion (ΔS_f_). Compounds that remained fully amorphous at the last time point (*t* = 168 days) were classified as stable, and compounds that crystallized partly or completely at any time point between 0 and 168 days were considered unstable.

We also investigated the possible association of the physicochemical properties with the long-term physical stability of the studied compounds subjected to different humidity levels. The relationships between the physicochemical properties and the long-term dry stability of the spray-dried amorphous compounds were quite similar to those observed for the GFA (Figure 7 and Figure S5), except that fewer of the compounds remained stable under humid conditions than under dry conditions. It was noticed that similar physicochemical properties seem to be important for GFA and stability, but it also became clear that compounds with fairly similar physicochemical properties can act entirely differently with regard to GFA and physical stability. Indapamide and prednisone have comparable physicochemical properties, but the former showed good GFA and excellent long-term physical stability under humid conditions compared to the latter. Similarly, sulfathiazole was more physically stable under dry conditions, whereas sulfamerazine with similar physicochemical properties was a nGF under the conditions of the spray-drying. Based on this dataset, PSA also came out as an indicator of good physical, dry stability—the higher the PSA, the greater the likelihood it would be a stable glass. From our findings, it is clear that good GFA does not necessarily correlate with good long-term physical stability, and cannot be predicted from any individual physicochemical properties, especially if the compound is exposed to humidity.

#### 3.5.3. Physicochemical Properties of Importance to GFA and Physical Stability

The observations from our study are in reasonable agreement with those reported in other studies. Previous studies have shown a correlation between long-term amorphous stability (assessed by determining the onset of crystallization) and several physicochemical properties including MW, HBD, heavy atom counts, T_g_, and ΔH_f_ [22]. While high MW, HBD, heavy atom count and T_g_ were more likely to have a beneficial impact on the physical stability, ΔH_f_ negatively influenced amorphous stability.

The role of T_rg_ in promoting amorphous formation and stability is also commonly emphasized. It is the ratio of T_g_/T_m_ that explains the viscosity of the amorphous system between the glass and melted phases. The higher the T_rg_, the higher the viscosity and thus the better the GFA and physical stability (lower crystallization tendency) [29,30]. From our study, compounds with higher T_rg_ had better GFA and physical stability. A contradicting finding, however, has been described by Baird et al. [31]; compounds with similar T_rg_ and melt viscosity (e.g., chlorpropamide and ketoconazole) showed different propensities for crystallization. This was explained by the different rates at which the viscosity changes with the change in temperature [31].

Another interesting area, but one not well explored, is the role of T_c_ in GFA and GS. It appears to be related to physical stability [10]; the higher the T_c_, the lower the crystallization tendency. Although there was a tendency for compounds with high T_c_ in our dataset to have relatively better physical stability, we did not obtain as strong a correlation as has previously been observed [10].

There has, to date, been no clear contribution of T_m_ reported in the literature in this respect [22,32]. Although a high T_m_ is sometimes associated with a high ΔH_f_, we demonstrated a weak positive relationship between T_m_ and GFA and/or physical stability in our study. This can be explained to some extent by the relationships between T_m_, T_g_ and T_c_, where compounds with higher T_m_ tend to have higher T_g_ and T_c_. To test this hypothesis, we performed a linear regression analysis to find out if T_m_ was linearly correlated with T_c_ and T_g_. The outcome of the analysis showed a modest linear relationship between T_m_ and both T_c_ and T_g_, with average *R*^2^ values of 0.67 and 0.71, respectively (Figure S6). Additionally, from our dataset, T_m_ was not correlated with ΔH_f_ (Figure S7; *R*^2^ < 0.1).

Taking into account the substantial effect of the preparation method on the properties of amorphous compounds, our attempt to find a possible correlation between the GFA and physical stability of the compounds was partly limited by the fact that some physicochemical properties (i.e., T_g_ and T_c_) used for the univariate and multivariate analyses were obtained from melt-quenched samples instead of from spray-dried samples, while the actual stability study was performed on spray-dried amorphous samples. With the spray-drying process parameters used in this study, only 50% formed an amorphous substance at all; thus, the values of these two properties were not measurable for the remaining compounds that were spray-dried as crystalline. Therefore, melt-quenched values were used instead. If the measured values and outcomes from the stability study were obtained from amorphous compounds prepared via the same method, a better relationship might have been discovered.

## 4. Conclusions

Based on a relatively large dataset (*n* = 26), this study shows that the GFA/GS classification and long-term physical stability are significantly influenced by the choice of method of preparation. The observed differences were attributed to many compounds having T_g_ and T_c_ too close to the outlet temperature and, to some extent, specific interactions with the solvents used upon spray-drying. From a pharmaceutical formulation development perspective, it is therefore critical to make sure that the method used for the GFA/GS group screening is similar to the method that will be used for manufacturing the final product.

The predictions of storage stability of spray-dried compounds from GFA/GS classification seem to be feasible. If the spray-dried material has been assigned to Class III, it is highly likely to be stable on storage at dry conditions for at least six months. For Class II compounds, the T_c_ is critical to evaluate as the amorphous compounds with T_c_ above about 120 °C were stable over the full storage time whereas compounds with lower T_c_ crystallized at the same storage conditions.

Selection of potential candidates for amorphous-based formulations may also be assisted by knowing important physicochemical properties. In this study, we confirmed that MW, T_c_, T_g_, T_rg_, ΔH_f_, and ΔS_f_ influence the GFA.

In addition, this study gives further support to the vital role of water in inducing and accelerating the crystallization process and thus lowering the stability of amorphous compounds. The stability of amorphous materials at humid conditions seem to be governed by specific solid phase–water interactions. Therefore, a better understanding of the role of water in GS must be attained to enable predictions of storage stability at humid conditions.

## Figures and Tables

**Table 1:** Model compounds and their respective physicochemical properties and glass-forming ability classes via melt-quenching and spray-drying method.

Compound	GFA MQ	GFASD	MW (g/mol)	logP	HBD	HBA	RotB	PSA (Å^2^)	T_g_ MQ (°C) ^a^	T_g_ SD (°C) ^b^	T_c_ MQ (°C) ^c^	T_c_ SD (°C) ^d^	T_m_ (°C)	T_rg_	ΔH_f_ (J/g)	ΔS_f_ (Jg^−1^K^−1^)
Acetaminophen	II	I	151	0.5	2	2	1	49	27	n.d	78	n.d	166	0.16	206	0.47
Aripiprazole	II	II	448	4.6	1	4	7	45	37	31	91	63	136	0.27	92	0.22
Bezafibrate	III	I	362	3.8	2	4	7	76	42	n.d	n.d.	n.d	182	0.23	145	0.32
Chlorpropamide	II	I	277	2.3	2	3	4	84	19	n.d	62	n.d	127	0.15	101	0.25
Cinnarizine	II	I	369	5.8	0	2	6	7	10	n.d	78	n.d	117	0.09	118	0.30
Clofoctol	II	I	365	8.1	1	1	5	20	−1	n.d	48	n.d	85	−0.01	98	0.27
Clotrimazole	III	II	345	5	0	1	4	18	33	15	n.d.	88	141	0.23	101	0.24
Droperidol	II	II	379	3.5	1	4	6	53	36	33	117	73	149	0.24	103	0.24
d-salicin	II	I	286	−1.2	5	7	4	120	62	n.d	129	n.d	198	0.31	201	0.43
Fenofibrate	III	I	361	5.2	0	4	7	53	−16	n.d	n.d.	n.d	79	−0.20	98	0.28
Flurbiprofen	II	I	244	4.2	1	3	3	37	−2	n.d	38	n.d	111	−0.02	116	0.30
Glibenclamide	III	II	494	4.8	3	5	8	122	61	75	n.d.	130	171	0.36	119	0.27
Glipizide	II	II	446	1.9	3	6	7	139	62	64	112	68	200	0.31	208	0.44
Hydrochlorothiazide	III	II	298	−0.1	3	7	1	135	119	76	n.d.	125	264	0.45	122	0.23
Hydrocortisone	III	II	362	1.6	3	5	2	95	93	96	n.d.	137	216	0.43	121	0.25
Ibuprofen	III	I	206	3.5	1	2	4	37	−40	n.d	n.d.	n.d	73	−0.55	135	0.39
Indapamide	III	III	366	2.9	2	5	3	101	107	106	n.d.	n.d	163	0.66	74	0.17
Ketoconazole	III	II	531	4.3	0	6	7	69	48	43	n.d.	104	145	0.33	103	0.25
Ketoprofen	III	I	254	3.1	1	3	4	54	0	n.d	n.d.	n.d	91	−0.01	114	0.31
Metolazone	III	II	366	4.1	2	5	2	101	121	137	n.d.	194	261	0.46	96	0.18
Prednisone	III	II	358	1.5	2	5	2	92	137	96	n.d.	92	186	0.74	95	0.21
Probucol	III	II	517	11.3	2	4	8	91	29	26	n.d.	44	123	0.24	68	0.17
Procaine	III	I	236	1.9	1	4	7	56	−36	n.d	n.d	n.d	58	−0.62	110	0.33
Sulfamerazine	III	I	264	0.1	2	6	3	106	69	n.d	n.d.	n.d	233	0.30	154	0.30
Sulfathiazole	III	II	255	0.1	2	6	3	122	65	51	n.d.	71	197	0.33	117	0.25
Tinidazole	II	I	247	−0.4	0	5	4	106	−7	n.d	53	n.d	123	−0.06	145	0.37

^a^ T_g_ for the substance formed by MQ; ^b^ T_g_ for the substance formed by SD; ^c^ T_c_ for the substance formed by MQ; ^d^ T_c_ for the substance formed by SD. GFA = glass forming ability; HBA = hydrogen bond acceptors; HBD = hydrogen bond donors; ΔH_f_ = enthalpy of fusion; logP = partition coefficient between octanol and water; MW = molecular weight; MQ = melt-quenching; PSA = polar surface area; RotB = number of rotatable bonds; ΔS_f_ = entropy of fusion; T_c_ = crystallization temperature; T_g_ = glass transition temperature; T_m_ = melting temperature; T_rg_ = reduced glass transition temperature; SD = spray-drying; n.d. = not detectable.

## Supplementary Materials

The following are available online at https://www.mdpi.com/1999-4923/11/9/425/s1, Figure S1: The change in intensity of PXRD diffractograms of a spray-dried sulfathiazole sample, analyzed twice in the PXRD instrument with 20 min between runs., Figure S2: The PLM micrographs of the freshly spray-dried sulfathiazole immediately after overnight drying (left) and after taken out for 2 h and exposed to ambient condition during various solid-state analyses (right)., Figure S3: Univariate analysis of the relationships between the glass-forming ability of the compounds upon spray-drying and selected physicochemical properties., Figure S4: Univariate analysis of the relationships between the glass-forming ability of the compounds upon spray-drying and selected physicochemical properties., Figure S5: Univariate analysis of the relationships between the long-term physical stability of the spray-dried compounds stored for 168 days (6 months) under humid conditions (75% RH) and selected physicochemical properties., Figure S6: Linear regression analysis plot showing the relationship between melting point (T_m_) and (**a**) crystallization temperature (T_c_) and (**b**) glass transition temperature (T_g_).The T_c_ and T_c_ are positively correlated with T_m_ (*R*^2^ = 0.67 and 0.71, respectively)., Figure S7: Linear regression analysis plot showing the relationship between melting point (T_m_) and heat of fusion (ΔH_f_). No correlation between ΔH_f_ and T_m_ was observed (*R*^2^ = 0.08). Table S1: Time to start and complete crystallization and the fraction of amorphous crystallized throughout the stability study period.
