# Supplementary material for: Long-Term Physical (In)Stability of Spray-Dried Amorphous Drugs: Relationship with Glass-Forming Ability and Physicochemical Properties
Source: Pharmaceutics. 2019 Aug 21;11(9):425. doi: 10.3390/pharmaceutics11090425 (PMC6781026; doi:10.3390/pharmaceutics11090425)
Supplement: Supplementary file 1 [file pharmaceutics-11-00425-s001.pdf]

pharmaceutics

Supplementary Materials: Long-Term Physical (In)Stability of Spray-Dried Amorphous Drugs: Relationship with Glass-Forming Ability and Physicochemical Properties

Khadijah Edueng, Christel A.S. Bergström, Johan Gråsjö and Denny Mahlin

Table S1. Time to start and complete crystallization and the fraction of amorphous crystallized throughout the stability study period.

Compound	Time to start crystallization (day)		Time to complete crystallization (day)		Fraction of amorphous crystallized (f_{CR}) at the last sampling point (168 days)		Remark	Polymorphism
	Dry (D)	Humid (H)	Dry (D)	Humid (H)	Dry (D)	Humid (H)		
Indapamide	>168	>168	>168	>168	0	0	Spray dried as fully amorphous<br>D: Remained 100% amorphous at the end of study<br>H: Remained 100% amorphous at the end of study	No
Metolazone	>168	>168	>168	>168	0	0	Spray dried as fully amorphous<br>D: Remained 100% amorphous at the end of study<br>H: Remained 100% amorphous at the end of study	No
Glibenclamide	>168	>0 ; ≤ 1	>168	>168	0	0.06	Spray dried as fully amorphous<br>D:Remained 100% amorphous at the end of study<br>H: Crystallization started between 0 and 1 day and progressed very slowly; still mainly amorphous after 168 days	Yes
Hydrocortisone	>168	>0 ; ≤ 1	>168	>168	0	0.11	Spray dried as fully amorphous<br>D: Remained completely amorphous at the end of the study<br>H: Crystallization started between 0 and 1 day and progressed very slowly; still mainly amorphous after 168 days	Yes
Hydrochlorothiazide	>168	>0 ; ≤ 1	>168	>0 ; ≤ 1	0	1	Spray dried as fully amorphous<br>D: Remained 100% amorphous at the end of the study<br>H: Fast crystallization; crystallization started and completed between 0 and 1 day	Yes
Ketoconazole	>1 ; ≤ 2	>0 ; ≤ 1	>168	>2 ; ≤ 7	1	1	Spray dried as fully amorphous<br>D: Crystallization started between 1 and 2 days to produce 100% crystalline at the end of the study<br>H: Crystallization started between o and 1 days and completed between 2 and 7 days	No
Sulfathiazole	>14; ≤ 28	>0 ; ≤ 1	>168	>0 ; ≤ 1	0.38	1	Spray dried as fully amorphous<br>D: Crystallization started between 14 and 28 days and progressed very slowly to produce 2% crystalline at the end of the study<br>H: Fast crystallization; crystallization started and completed between 0 and 1 day	Yes
Prednisone	>28; ≤ 84	>0; ≤ 1	>168	>0; ≤ 1	0.07	1	Some crystalline already present in freshly spray dried sample<br>D: Slow crystallization; crystallization started between 28 and 84 days to produce 7% crystalline compared to fresh sample at the end of the study<br>H: Crystallization started and completed between 0 and 1 day to produce 100% crystalline	Yes
Aripiprazole	>28; ≤ 84	>0; ≤ 1	>168	>2; ≤ 7	0.15	1	Some crystalline already present in freshly spray dried sample<br>D: Slow crystallization; crystallization of amorphous content started between 28 and 84 days to produce 2 % crystalline compared to fresh sample at the end of the study<br>H: Crystallization of amorphous content started between 0 and 1 day but completed between 2 and 7 days	Yes
Glipizide	>0; ≤ 1	>0; ≤ 1	>168	>1; ≤ 2	0.08	1	Some crystalline already present in the freshly spray dried sample	Yes

Compound	Time to start crystallization (day)		Time to complete crystallization (day)		Fraction of amorphous crystallized (f_{CR}) at the last sampling point (168 days)		Remark	Polymorphism
	Dry (D)	Humid (H)	Dry (D)	Humid (H)	Dry (D)	Humid (H)		
							D: Crystallization of amorphous content started between 0 and 1 day but not completed until the end of the study (89% crystalline produced)<br>H: Crystallization of amorphous content started between 0 and 1 day but completed between 1 and 2 days	
Droperidol	>84; ≤ 168	>0; ≤ 1	>168	>0; ≤ 1	0.04	1	Some crystalline already present in the freshly spray dried sample<br>D: Slow crystallization; crystallization of amorphous content started between 84 and 14 days to produce 6 % crystalline compared to fresh sample at the end of the study<br>H: Crystallization of amorphous content started and completed between 0 and 1 day	Yes
Clotrimazole	>2; ≤7	>2; ≤ 7	>7; ≤ 14	>2; ≤ 7	1	1	Some crystalline already present in the freshly spray dried sample<br>D: Crystallization of amorphous content started between 2 and 7 days and completed between 7 and 14 days<br>H: Crystallization of amorphous content started and completed between 2 and 7 days	No
Probucol	>0; ≤ 1	>0; ≤ 1	>2; ≤ 7	>2; ≤ 7	1	1	Some crystalline already present in the freshly spray dried sample<br>D: Crystallization of amorphous content started between 0 and 1 day and completed between 2 and 7 days<br>H: Crystallization of amorphous content started between o and 1 day and completed between 2 and 7 days	Yes
Acetaminophen	NA	NA	NA	NA	NA	NA	Completely crystalline upon spray drying	No
Bezafibrate	NA	NA	NA	NA	NA	NA	Completely crystalline upon spray drying	Yes
Chlorpropamide	NA	NA	NA	NA	NA	NA	Completely crystalline upon spray drying	Yes
Cinnarizine	NA	NA	NA	NA	NA	NA	Completely crystalline upon spray drying	No
Clofoctol	NA	NA	NA	NA	NA	NA	Completely crystalline upon spray drying	Yes
D-Salicin	NA	NA	NA	NA	NA	NA	Completely crystalline upon spray drying	Yes
Fenofbirate	NA	NA	NA	NA	NA	NA	Completely crystalline upon spray drying	No
Flurbiprofen	NA	NA	NA	NA	NA	NA	Completely crystalline upon spray drying	Yes
Ibuprofen	NA	NA	NA	NA	NA	NA	Completely crystalline upon spray drying	No
Ketoprofen	NA	NA	NA	NA	NA	NA	Completely crystalline upon spray drying	No
Procaine	NA	NA	NA	NA	NA	NA	Completely crystalline upon spray drying	No
Sulfamerazine	NA	NA	NA	NA	NA	NA	Completely crystalline upon spray drying	Yes
Tinidazole	NA	NA	NA	NA	NA	NA	Completely crystalline upon spray drying	Yes

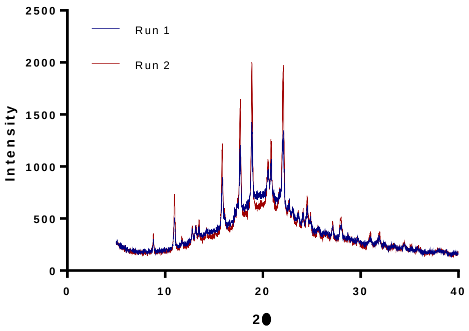

Figure S1. The change in intensity of PXRD diffractograms of a spray-dried sulfathiazole sample analysed twice in the PXRD instrument. The second run (Run 2) was performed 20 min after the first run (Run 1). The diffractogram of the second run (Run 2) shows an increase in the intensity of the peak indicating increase in crystallinity between the two runs.

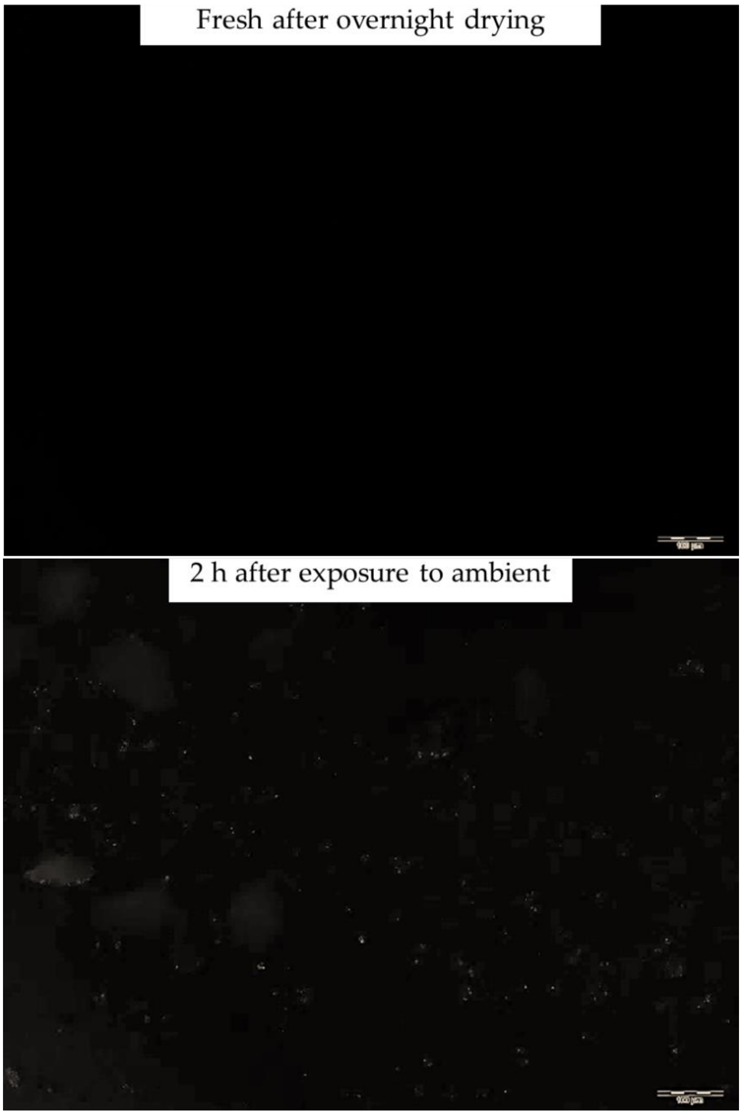

Figure S2. The PLM micrographs of the freshly spray-dried sulfathiazole immediately after overnight drying (top) and after taken out for 2 h and exposed to ambient condition during various solid state analyses (right). Birefringence observed in the PLM micrograph (bottom) of sulfathiazole after 2 h exposure to ambient condition.

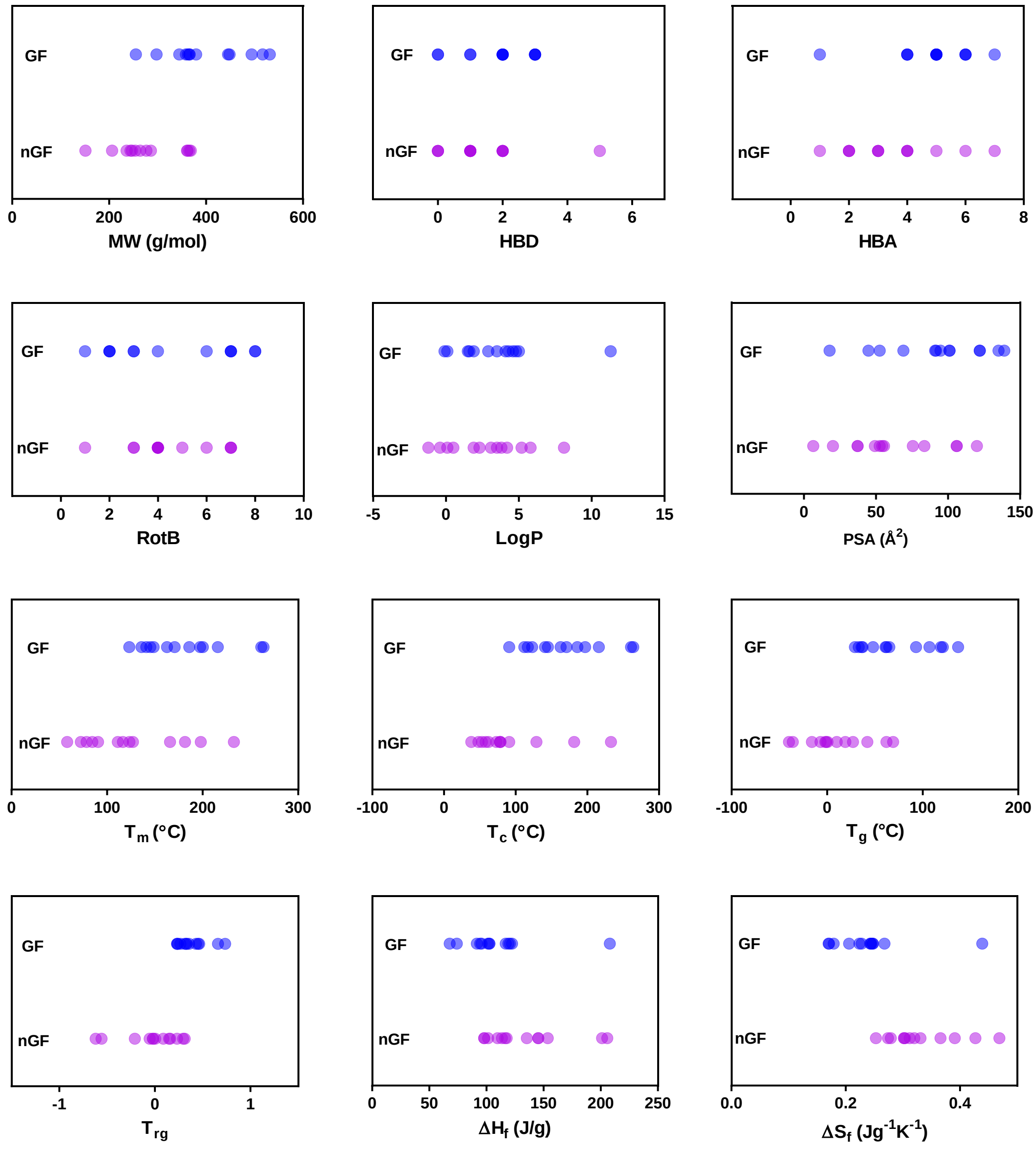

Figure S3. Univariate analysis of the relationships between the glass-forming ability of the compounds upon spray drying and selected physicochemical properties. The data are shown for the following physicochemical properties: molecular weight (MW), number of hydrogen bond donors (HBD), number of hydrogen bond acceptors (HBA), number of rotatable bonds (RotB), log*P*, polar surface area (PSA), melting point (T_m), crystallization temperature (T_c), glass transition temperature (T_g), reduced glass transition temperature (T_{rg}), heat of fusion (ΔH_f) and entropy of fusion (ΔS_f). The compounds are classified as either glass formers (GFs) or non-glass formers (nGFs).

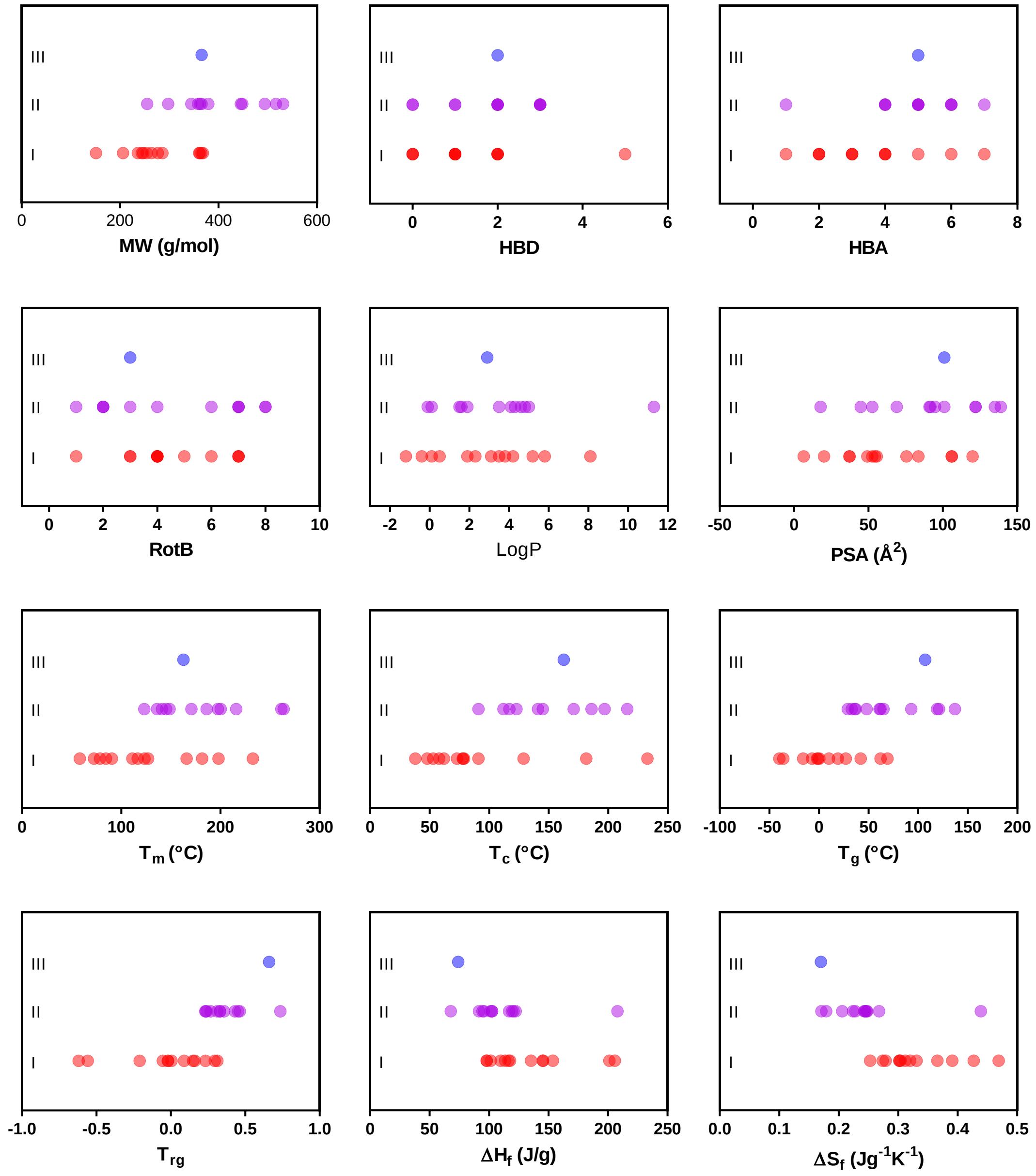

Figure S4. Univariate analysis of the relationships between the glass-forming ability of the compounds upon spray drying and selected physicochemical properties. The data are shown for the following physicochemical properties: molecular weight (MW), number of hydrogen bond donors (HBD), number of hydrogen bond acceptors (HBA), number of rotatable bonds (RotB), log*P*, polar surface area (PSA), melting point (T_m), crystallization temperature (Tc), glass transition temperature (T_g), reduced glass transition temperature (T_{rg}), heat of fusion (ΔH_f) and entropy of fusion (ΔS_f). The compounds are classified as either stable glass formers (Class I), unstable glass formers (Class II) and non-glass formers (Class I).

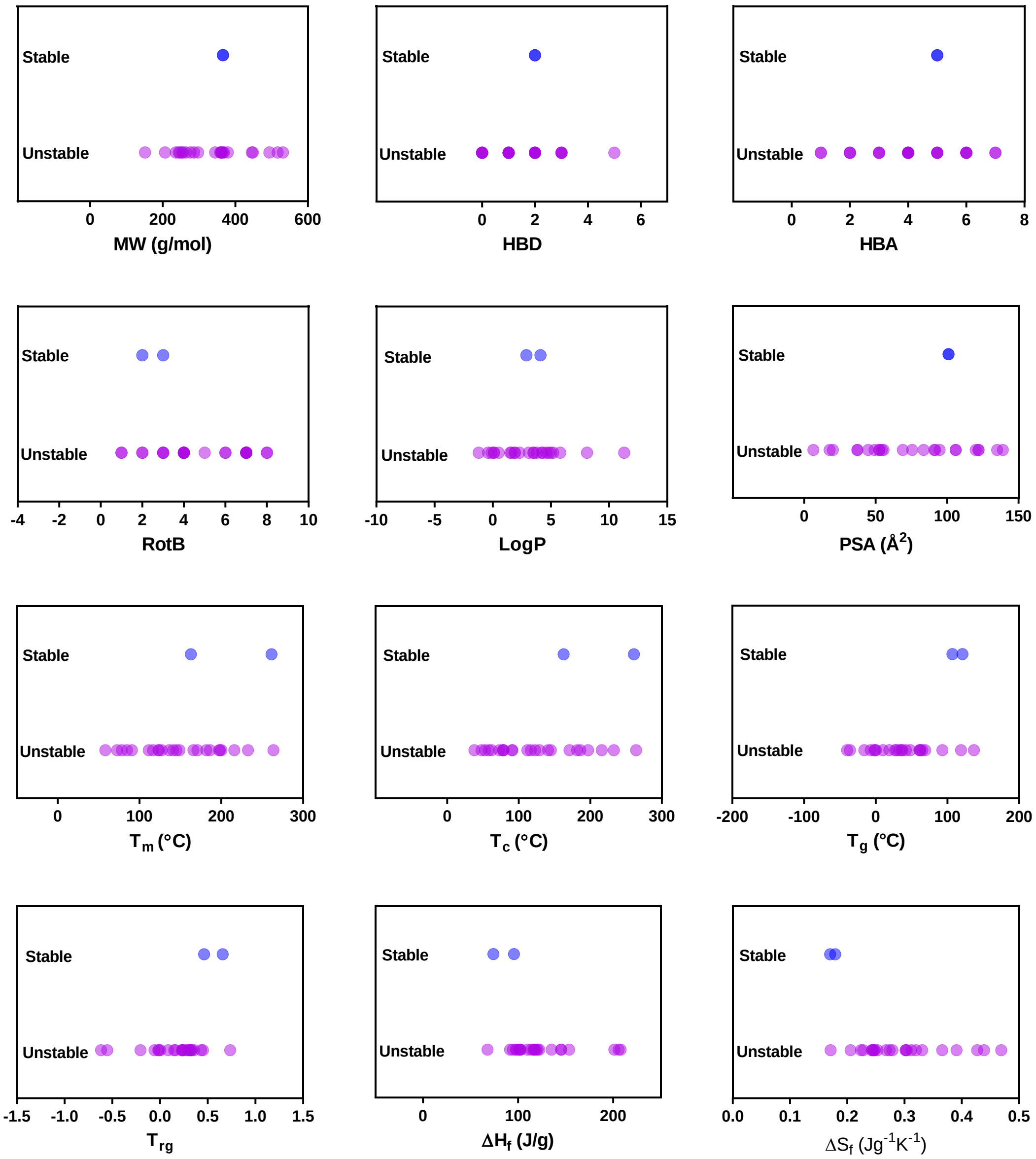

Figure S5. Univariate analysis of the relationships between the long-term physical stability of the spray-dried compounds stored for 168 days (6 months) under humid conditions (75% RH) and selected physicochemical properties. The physicochemical properties included were molecular weight (MW), number of hydrogen bond donors (HBD), number of hydrogen bond acceptors (HBA), number of rotatable bonds (RotB), log*P*, polar surface area (PSA), melting point (T_m), crystallization temperature (T_c), glass transition temperature (T_g) and reduced glass transition temperature (T_{rg}), heat of fusion (ΔH_f) and entropy of fusion (ΔS_f). Compounds that remained fully amorphous at the last time point (t = 168 days) were classified as stable and compounds that crystallized partly or completely at any time point between 0 and 168 days were considered unstable.

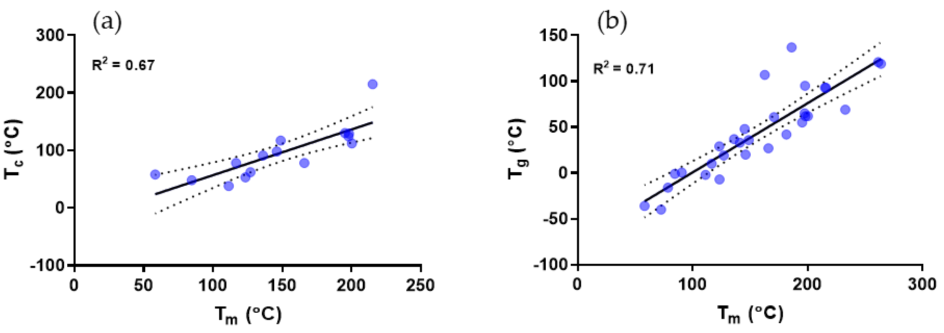

Figure S6. Linear regression analysis plot showing the relationship between melting point (T_m) and (**a**) crystallization temperature (T_c) and (**b**) glass transition temperature (T_g). The black line represents the linear fit of the dataset and the dotted lines represent the 95% confidence intervals. Only compounds with detectable and measurable T_c and T_g were included in this analysis. The T_c and T_c are positively correlated with T_m (R^2 = 0.67 and 0.71, respectively).

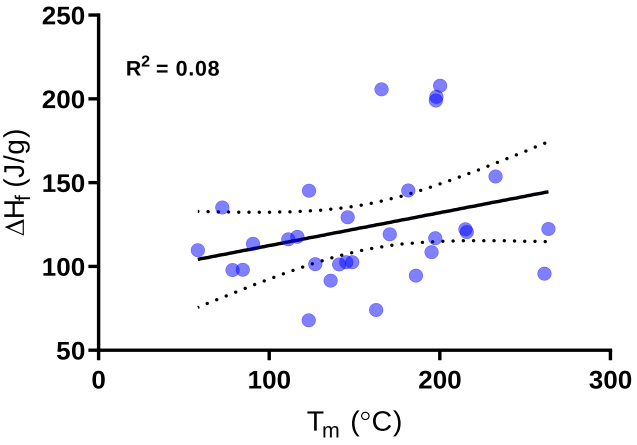

Figure S7. Linear regression analysis plot showing the relationship between melting point (T_m) and heat of fusion (ΔH_f). The black line represents the linear fit of the dataset and the dotted lines represent the 95% confidence intervals. Only compounds with detectable and measurable T_c and T_g were included in this analysis. No correlation between ΔH_f and T_m was observed (R^2 = 0.08).
